# Supplementary material for: Improving Stuttering Through Augmented Multisensory Feedback Stimulation
Source: Brain Sci. 2025 Feb 25;15(3):246. doi: 10.3390/brainsci15030246 (PMC11939842; doi:10.3390/brainsci15030246)
Supplement: Supplementary file 1 [file brainsci-15-00246-s001.zip › brainsci-3470491-supplementary.pdf]

Improving Stuttering through the Augmented Multisensory Feedback Stimulation: protocol presentation and validation

Supplementary materials

The following tables summarize the physiological signals measured at the three different time points for the patients and in one registration in healthy control participants.

Table S1 – SSI-4 parameters and total scores

Parameters	T0	T1	T2	Within-group comparison	Control group	Between-group comparison
**Frequency**	10.50 [8.00, 13.00]	0.00 [0.00, 2.00]	0.00 [0.00, 0.00]	**p < .001 T0<T1: <.001 T0<T2: <.001**	0.00 [0.00, 0.50]	**T0 vs HC < .001**
**Duration**	3.00 [2.00, 5.75]	1.00 [0.00, 1.00]	0.00 [0.00, 0.75]	**p < .001 T0<T1: <.001 T0<T2: <.001**	0.00 [0.00, 1.00]	**T0 vs HC <.001** T1 vs HC = .071
**Physical concomitants**	1.00 [0.00, 2.88]	0.00 [0.00, 0.00]	0.00 [0.00, 0.00]	**p < .001 T0<T1: <.001 T0<T2: <.001**	0.00 [0.00, 0.00]	**T0 vs HC <.001**
**Total scores**	15.75 [11.00, 21.00]	1.00 [0.00, 2.75]	0.00 [0.00, 0.75]	**p < .001 T0<T1: <.001 T0<T2: <.001**	0.00 [0.00, 1.50]	**T0 vs HC <.001**

Table S1 summarizes the descriptive values (median and interquartile range) of parameters and total scores during the rest conditions at the three different time points for the patients' group and the only rest condition in the control group. Significant comparisons within and between groups (columns five and seven, respectively) are boldly highlighted.

Table S2 – descriptive values at the resting state conditions

Variables	T0	T1	T2	Within-group comparison	Control group	Between-group comparison
**BVP (mean beats min)**	83.32 [73.70, 90.38]	74.54 [65.93, 86.00]	74.81 [66.94, 85.47]	**p < .001 T0 > T1 = .013 T0 > T2 = .001**	78.25 [70.13, 86.22]	ps >. 495
**EMG (mean uV)**	2.33 [1.99, 3.36]	1.98 [1.77, 2.70]	2.32 [1.66, 3.23]	p = .130	2.05 [1.85, 2.48]	ps >. 246
**SC (mean uS)**	3.67 [1.95, 6.89]	3.45 [1.76, 5.05]	3.04 [1.66, 4.55]	p = .126	2.60 [1.23, 3.86]	ps >. 100
**Temperature (mean Degrees)**	30.20 [27.44, 32.52]	29.81 [27.41, 32.61]	32.13 [29.66, 33.21]	**p = .010 T1 < T2 = .011**	29.10 [24.99, 31.14]	**T2 > HC = .004**
**Respiration Rate (mean breaths/ minute)**	14.66 [12.88, 16.36]	15.41 [13.17, 16.88]	15.70 [14.09, 17.23]	p = .080	15.94 [14.46, 16.65]	ps > .304

Table S2 summarizes the descriptive values (median and interquartile range) of physiological indexes during the rest conditions at the three different time points for the patients' group and the only rest condition in the control group. Significant comparisons within and between groups (columns five and seven, respectively) are boldly highlighted.

Table S3 – descriptive values for the spontaneous speech conditions

Variables	T0	T1	T2	Within-group comparison	Control group	Between-group comparison
**BVP (mean beats min)**	94.14 [85.26, 103.45]	86.53 [74.80, 94.15]	82.68 [75.85, 90.82]	**p < .001 T0>T1: < .001 T0>T2: < .001**	101.66 [86.45, 106.75]	**T1 < HC = .030 T2 < HC = .005**
**EMG (mean uV)**	5.40 [3.69, 8.79]	3.48 [2.79, 4.94]	3.59 [3.08, 5.06]	**p < .001 T0>T1: < .001 T0>T2 = .001**	5.42 [4.66, 8.55]	**T1 < HC: < .001 T2 < HC: < .001**
**SC (mean uS)**	6.80 [2.97, 10.01]	5.44 [2.85, 9.99]	5.15 [2.17, 7.20]	p = .306	4.41 [2.59, 5.75]	ps > . 118
**Temperature (mean Degree)**	30.34 [27.66, 33.36]	30.36 [27.16, 33.32]	32.38 [30.03, 33.47]	**p = .034 T1 < T2 = .042**	29.90 [25.50, 31.78]	**T2 > HC = .004**
**Respiration Rate (mean breaths/ minute)**	11.26 [9.98, 12.18]	11.29 [10.41, 12.45]	11.36 [10.47, 12.47]	p = .901	11.36 [10.65, 11.81]	ps = 1

Table S3 summarizes the descriptive values (median and interquartile range) of physiological indexes during the spontaneous speech conditions at the three different time points for the patients' group and the only spontaneous speech measurement in the control group. Significant comparisons within and between groups are boldly highlighted.

Table S4 – descriptive values for the reading-aloud conditions.

Variables	T0	T1	T2	Within-group comparison	Control group	Between-group comparison
**BVP (mean beats min)**	92.94 [84.06, 98.45]	82.53 [74.86, 94.05]	80.02 [69.40, 90.94]	**< .001**<br>**T0 > T1 = .004**<br>**T0 > T2: < .001**	90.61 [81.06, 102.62]	**T2 < HC = .021**
**EMG (mean uV)**	7.29 [4.94, 11.11]	3.62 [2.76, 6.05]	3.58 [2.74, 6.32]	**< .001**<br>**T0 > T1: < .001**<br>**T0 > T2: < .001**	6.08 [4.77, 10.44]	**T1 < HC = .002**<br>**T2 < HC = .003**
**SC (mean uS)**	8.04 [3.96, 13.35]	6.45 [3.00, 10.88]	5.24 [2.58, 8.40]	p = .063	4.72 [2.79, 6.34]	**T0 > HC = .018**
**Temperature (mean Degree)**	30.69 [28.23, 33.33]	30.88 [27.53, 33.82]	32.67 [30.19, 33.76]	p = .063	28.84 [26.12, 31.68]	**T2 > HC = .003**
**Respiration Rate (mean breaths/ minute)**	11.50 [10.39, 12.51]	10.07 [8.92, 11.12]	9.30 [8.19, 10.23]	**<0.001**<br>**T0 > T1 = .004**<br>**T0 > T2: <.001**	11.99 [10.84, 13.10]	**T1 < HC: <.001**<br>**T2 < HC: <.001**

Table S4 summarizes the descriptive values (median and interquartile range) of physiological indexes during the reading conditions at the three different time points for the patients' group and the only spontaneous speech measurement in the control group. Significant comparisons within and between groups are boldly highlighted.

SSI-4 Individual differences

Individual trends in SSI-4 total scores have been graphically explored and are depicted in Figure S1.

The Spaghetti plot shows subjects with a disease score ≥ 9 at T0. A cutoff value of 9 was chosen, representing the third quartile of the disease variable, thus identifying the subgroup of patients with a worse disease condition. As can be seen, all subjects experienced an improvement at T1, whereas more heterogeneous patterns emerged at T2, with some participants showing enhanced improvements, others showing stable performance, and others worsening compared to T1.

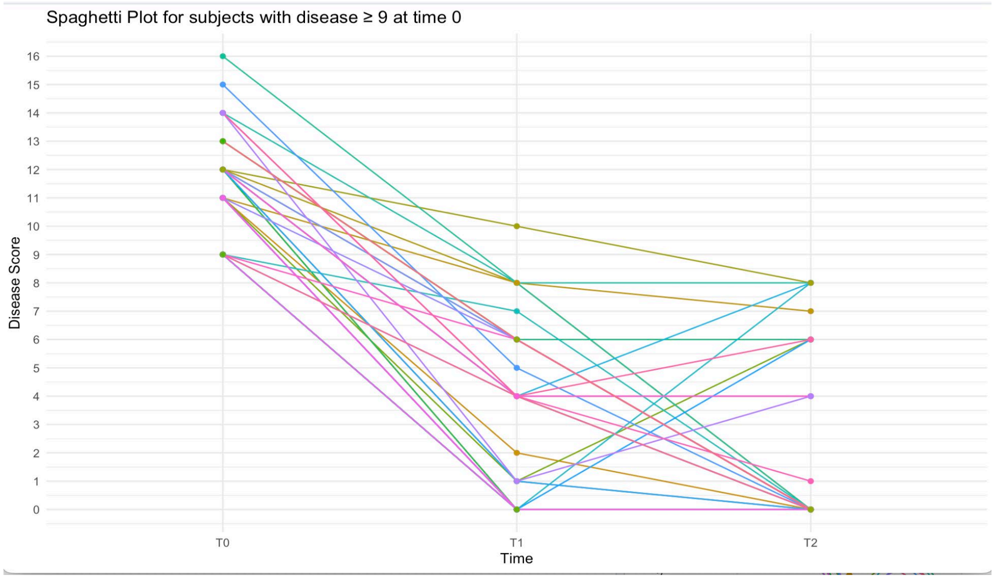

Figure S1 shows the subject trend in SSI-4 total scores at the three time points.

Impact of individual differences on disease score

A mixed-effects model was applied to explore the factors influencing the disease score over time, with severity at time 0, age, and gender as fixed predictors, and a random intercept for each subject to account for individual variability. As shown in Table 1, individuals with Low Severity at baseline had significantly lower disease scores than those with High Severity. Specifically, the coefficient for Severity was -8.87, indicating that the disease score was, on average, 8.87 points lower for individuals with lower baseline severity. This effect was significant ($p < .001$), suggesting a strong relationship between initial severity and the disease score. Age did not influence the disease score over time ($p = .599$). The effect of gender showed a trend toward significance ($p = .075$), probably due to the discrepancy between males (N = 40) and females (N = 6).

Table S5. Multivariable mixed model with random effects for patients

Variable	Estimate	p
Severity (Low vs Severity)	-8.87	<0.001
Age	-0.02	0.599
Male	1.93	0.075
